# Dietary supplementation with 25-hydroxycholecalciferol is more effective than cholecalciferol in improving reproductive performance in aged duck breeders

**DOI:** 10.1093/jas/skag037

**Published:** 2026-02-12

**Authors:** Yating Li, Yongyan Jin, Lei Zhuang, Wei Zhou, Shuaiqin Wang, Jindang Cao, Mingkai Wang, Li Chen, Jiannan Zhao, Zhengkui Zhou, Ming Xie, Shuisheng Hou, Jing Tang

**Affiliations:** State Key Laboratory of Animal Nutrition and Feeding, Institute of Animal Science, Chinese Academy of Agricultural Sciences, Beijing, 100193, China; State Key Laboratory of Animal Nutrition and Feeding, Institute of Animal Science, Chinese Academy of Agricultural Sciences, Beijing, 100193, China; State Key Laboratory of Animal Nutrition and Feeding, Institute of Animal Science, Chinese Academy of Agricultural Sciences, Beijing, 100193, China; State Key Laboratory of Animal Nutrition and Feeding, Institute of Animal Science, Chinese Academy of Agricultural Sciences, Beijing, 100193, China; State Key Laboratory of Animal Nutrition and Feeding, Institute of Animal Science, Chinese Academy of Agricultural Sciences, Beijing, 100193, China; Shandong Haineng Bioengineering Co., Ltd, Rizhao, 276800, China; Shandong Haineng Bioengineering Co., Ltd, Rizhao, 276800, China; Food Science and Engineering College, Beijing University of Agriculture, Beijing, 102206, China; State Key Laboratory of Animal Nutrition and Feeding, Institute of Animal Science, Chinese Academy of Agricultural Sciences, Beijing, 100193, China; State Key Laboratory of Animal Nutrition and Feeding, Institute of Animal Science, Chinese Academy of Agricultural Sciences, Beijing, 100193, China; State Key Laboratory of Animal Nutrition and Feeding, Institute of Animal Science, Chinese Academy of Agricultural Sciences, Beijing, 100193, China; State Key Laboratory of Animal Nutrition and Feeding, Institute of Animal Science, Chinese Academy of Agricultural Sciences, Beijing, 100193, China; State Key Laboratory of Animal Nutrition and Feeding, Institute of Animal Science, Chinese Academy of Agricultural Sciences, Beijing, 100193, China

**Keywords:** duck breeder, vitamin D_3_, 25-hydroxycholecalciferol, requirement, relative bioavailability

## Abstract

This study aimed to evaluate the effects of supplementing two vitamin D sources on reproductive performance, egg quality, and plasma biochemical indices of aged duck breeders, and to estimate vitamin D requirements and relative bioavailability from these two sources. A total of 180 laying Pekin ducks (64 wk of age) were randomly assigned to 9 different treatments, each containing 10 replicates with 2 birds per replicate. The birds were fed a basal diet with no vitamin D supplementation or supplemented with cholecalciferol (VD_3_) or 25-hydroxycholecalciferol (25-OH-D_3_) at 250, 500, 1000, or 2000 IU/kg of feed for 15 wk. The two-way ANOVA (2 × 4 factors, without a control group) and one-way ANOVA were employed to compare the differences between 25-OH-D_3_ and VD_3_. In comparison to ducks fed the basal diet, the egg production (9 to 15 wks), ovarian weight, and the number and weight of dominant follicles increased with increasing VD_3_ or 25-OH-D_3_ levels (linear and quadratic, *P *< 0.05). The egg Haugh unit exhibited a linear increase with rising VD_3_ levels (*P *= 0.021) and a quadratic increase with rising 25-OH-D_3_ levels (*P *= 0.042). Additionally, the plasma calcium content increased linearly as dietary 25-OH-D_3_ levels increased (*P *= 0.001). Furthermore, diets supplemented with 25-OH-D_3_ resulted in higher plasma 25-OH-D_3_ concentrations compared to those fed VD_3_ (*P *< 0.001). According to the quadratic broken-line model, the VD_3_ requirements of duck breeders in terms of enhancing egg production, ovarian weight, and dominant follicle number were 906, 359, and 730 IU/kg, respectively, whereas the 25-OH-D_3_ requirements were 260, 324, and 308 IU/kg, respectively. Based on slope ratio comparison from multiple linear regressions of ovarian weight, dominant follicle number, and plasma 25-OH-D_3_ concentration, the bioavailability of 25-OH-D_3_ were 147%, 191%, and 211%, respectively, relative to VD_3_.

## Teaser

Supplemental VD_3_ or 25-OH-D_3_ provided in the diets of duck breeders could increase the egg production, hatchability, Haugh unit, and 25-OH-D_3_ concentration in plasma. The efficacy of 25-OH-D_3_ in enhancing reproductive performance surpassed that of VD_3_. It is recommended to supplement the basal diet of duck breeders aged 64 to 70 wk with 906 IU/kg of VD_3_ or 324 IU/kg of 25-OH-D_3_, noting that the bioavailability of 25-OH-D_3_ relative to VD_3_ is 211%.

## Introduction

As a fat-soluble secosteroid, vitamin D_3_ (VD_3_) plays an important role in maintaining the metabolic of calcium (Ca) and bone formation in the body (Souberbielle et al. 2010; Zhuang et al. 2025). VD_3_ can be synthesized through exposure to ultraviolet B radiation from 7-dehydrocholesterol in the skin (Holick 2018). However, the rapid development of intensive farming models has caused receiving less ultraviolet B radiation in poultry, thereby limiting the endogenous synthesis of VD_3_ (Świątkiewicz et al. 2017). Therefore, dietary VD_3_ supplementation becomes critical for poultry.

The National Research Council (NRC 1994) proposed a dietary level of 900 IU/kg of VD_3_ for duck breeders; however, this recommendation lacks supporting literature. Furthermore, with the rapid advancements in breeding work, whether the NRC standards adequately address the current production of duck breeders is unknown. Diets for laying hens typically contain 2500 IU/kg VD_3_, exceeding the NRC recommendation (300 IU/kg) (Jing et al. 2022). Several studies have indicated that a high intake of VD_3_ improves egg production and the quality of eggshells in laying hens, especially in the later stages of the laying cycle (Kim et al. 2012; Wen et al. 2019). Similar to laying hens, breeding ducks also experience declining egg production rates and poor eggshell quality during the late laying phase (Cao et al. 2022; Jin et al. 2024), which reduces economic benefits and is detrimental to the growth of offspring. Consequently, investigating the VD_3_ requirements of duck breeders during the late laying phase is very meaningful, as it can fulfill the production needs of breeding ducks while preventing insufficient maternal VD_3_ sources in their offspring.

As a main circulating form of VD_3_, 25-hydroxycholecalciferol (25-OH-D_3_) is synthesized in the liver (Atencio et al. 2005). Since its identification in 2006 as an effective source of vitamin D (VD) for poultry, 25-OH-D_3_ has gained considerable traction and is now widely utilized in the poultry industry (Adhikari et al. 2020). It has been shown that adding 25-OH-D_3_ to the feed instead of VD_3_ can increase the egg production, egg quality and follicle development of laying hens (Geng et al. 2018; Hrabia et al. 2023; Gao et al. 2024; Jin et al. 2024). Additionally, it has been established that 25-OH-D_3_ is nearly twice as effective as VD_3_ in promoting not only egg production but also in contributing to bone mineralization and strength (Atencio et al. 2005; Han et al. 2016; Zhou et al. 2022). Nevertheless, Jing et al. (2022) suggested that adding 125 μg/kg of either VD_3_ or 25-OH-D_3_ to the diet had the same effect on laying performance and shell quality of hens (60 to 72 wk of age). These reports showed that there are differences in the comparisons of 25-OH-D_3_ and VD_3_ in different doses, and response variables. During the late laying period of meat-type breeder ducks, the effects of 25-OH-D_3_ relative to VD_3_ remain unclear. Therefore, this study aims to investigate the relative bioavailability (RBV) of 25-OH-D_3_ compared to VD_3_, as well as to evaluate their respective impacts on reproductive performance, egg quality, ovarian follicle development, and plasma biochemical indices in meat-type breeding ducks during the late laying period.

## Materials and methods

All experimental procedures in this study were approved by the Animal Care and Use Committee of the Institute of Animal Science, Chinese Academy of Agricultural Sciences (permission number: IAS2024-27). Furthermore, all procedures were meticulously conducted in accordance with the guidelines established for animal experimentation by the National Institute of Animal Health.

### Animals, diets, and experimental design

A total of 180 healthy 64-week-old laying duck breeders with similar body weight and egg production were randomly divided into 9 dietary groups, each with 10 replicates of 2 ducks. The dietary treatments included a basal diet without VD_3_ supplementation, a basal diet with VD_3_ supplementation at 250, 500, 1000, and 2000 IU/kg, and a basal diet with 25-OH-D_3_ supplementation at 250, 500, 1000, 2000 IU/kg, respectively. The basal diet was comprised of corn and soybean meal (Table 1). The VD_3_ used was procured from Zhejiang NHU Co., Ltd., Zhejiang, China, and 25-OH-D_3_ is 1.25 g/kg was obtained from Haineng Bioengineering Co., Ltd., Shandong, China. The trial period was 15 wk (64 to 79 wk of age).

**Table 1:** Composition and nutrient levels of the basal diet (air-dry basis).

Item	Content
**Ingredient, %**	
**Corn**	61.05
**Soybean meal**	30.00
**Calcium hydrophosphate**	1.50
**Limestone**	6.00
**Premix^a^**	1.00
**NaCl**	0.30
**DL-Methionine**	0.15
**Total**	100.00
**Calculated nutrient value, %**	
**AME^b^, Mcal/kg**	2.76
**Crude protein**	18.51
**Calcium**	2.80
**Nonphytate phosphorus**	0.40
**Lysine**	0.95
**Methionine**	0.45
**Methionine + cysteine**	0.78
**Threonine**	0.76
**Tryptophan**	0.23
**Arginine**	1.20

a Provided per kilogram of diet: Cu (CuSO_4_•5H_2_O), 10 mg; Fe (FeSO_4_•7H_2_O), 60 mg; Zn (ZnO), 60 mg; Mn (MnSO_4_•H_2_O), 80 mg; Se (NaSeO_3_), 0.3 mg; I (KI), 0.2 mg; choline chloride, 1,000 mg; vitamin A (retinyl acetate), 10,000 IU; vitamin E (DL-α-tocopheryl acetate), 20 IU; vitamin K_3_ (menadione sodium bisulfate), 2 mg; thiamin (thiamin mononitrate), 2 mg; riboflavin, 10 mg; pyridoxine hydrochloride, 4 mg; cobalamin, 0.02 mg; calcium-D-pantothenate, 20 mg; nicotinic acid, 50 mg; folic acid, 1 mg; biotin, 0.2 mg.

b Average metabolizable energy (AME) The value is calculated according to the AME of ducks.

Each duck breeder was housed in a cage individually (1.10 m length × 0.90 m width × 0.70 m height). At the same time, a total of 36 healthy male ducks were selected for mating, maintaining a male-to-female ratio of 1:5. During the mating period, the male and female ducks cohabited and fed together. They had unlimited access to feed and water, and constant lighting was maintained.

### Sample preparation and data collection

At the commencement of the study, all duck breeders were weighted. Food consumption was recorded throughout the study, and the average daily feed intake (ADFI) was calculated. Daily collection and weighing of eggs from each replicate were carried out, and these eggs are consistently kept at a temperature of 17 °C with a relative humidity of 75%. Throughout the 2nd, 4th, 6th, 8th, and 10th weeks of the experiment, 12 eggs from each replicate were chosen for incubation in a commercial incubator (Yiai 12096, Qingdao, China). The incubation temperature was maintained at 38.0 °C for days 1 to 7, at 37.7 °C for days 8 to 14, and at 37.4 °C for days 15 to 26, with a relative humidity level of 65%. On day 7 of incubation, we performed candling of the eggs to assess their fertility. Fertility was determined using the formula: (number of fertilized duck eggs/total number of incubated eggs) × 100%. On the 26th day after incubation, eggs were placed in hatcher at 36.5 °C and humidity 70%. On 28th day, the quantity and weight of ducklings hatched were recorded. The percentage of hatching of ducks was calculated using the following formula: (number of hatching ducks/total number of fertilized eggs) × 100%.

At the end of the study (at 79 wk of age), two ducks were randomly chosen from each replicate following a 12-h fasting period. Blood, totaling 5 mL, was extracted from the subwing vein of every duck, transferred into a heparin sodium anticoagulant tube, and centrifuged at 1520 × g for 10 mins at a temperature of 4 °C to obtain plasma. The obtained plasma was subsequently stored at −20 °C. Following slaughter, the ovaries of the ducks were weighed, and the ovarian index (ovarian weight/body weight) was calculated. Additionally, the number and weight of the dominant follicle and large yellow follicle were determined. The classification criteria for follicles, as previously described by Gilbert et al. (1980), indicated that a follicle diameter greater than 10 mm was classified as a dominant follicle, while a diameter between 8 and 10 mm was classified as a large yellow follicle.

### Measurements

Egg quality was measured using three average-weight eggs from each replicate for three consecutive days at the end of 15th wk, and all measurements completed on the collection day. The weight of the egg was recorded, the breaking strength was measured by egg force reader (Israel Orka Food Technology Ltd., Herzliya, Israel) and the eggshell thickness was taken with the help of eggshell thickness gauge (Tenovo International Co., Limited). The egg albumen height, Haugh unit, and yolk color were assessed using an egg analyzer (Tenovo International Co., Limited).

The levels of Ca and phosphorus (P) in plasma were evaluated using an automatic biochemical analyzer (Hitachi 7080, Tokyo, Japan) in conjunction with the applicable kits (Maccura Biotechnology Co. LTD, Chengdu, China). And the concentration of plasma 25-OH-D_3_ was assessed using liquid chromatography-tandem mass spectrometry (LC-MS, Xu et al., 2016). Prior to LC-MS analysis, 1.6 mL of methanol was added to 400 µL of plasma to precipitate proteins. After centrifugation at 14,000 rpm for 5 min, the supernatant was transferred to an Agilent filtration disk column (Captiva EMR-Lipid 96WP, Agilent). The column was activated with 1.6 mL of 80% methanol prior to sample addition, followed by drying under nitrogen gas. The residue was then reconstituted in 100 µL of methanol and analyzed by LC-MS to determine the content of 25-OH-D_3_.

### Statistical analysis

Each replicate served as an experimental unit. The effect of dietary supplementation with VD_3_ or 25-OH-D_3_ on each response variable was analyzed using a one-way ANOVA within the GLM procedure of SAS 9.4 (SAS Inst. Inc., Cary, NC). The linear and quadratic responses of the dependent variables to varying levels of VD_3_ or 25-OH-D_3_ were assessed using polynomial comparisons. The analysis was performed on data that did not include a basal diet lacking VD supplementation, utilizing a factorial design of 2 × 4 (sources × levels) for treatments, analyzed through a two-way ANOVA in the GLM procedure of SAS 9.4. The model considered the primary impacts of the source of VD as well as the level of added VD. Furthermore, a quadratic broken-line approach was employed to assess the ideal VD levels based on the response criteria (Robbins et al. 2006).

Utilizing VD_3_ as a reference source, a multiple linear regression approach centered on slope comparison was employed to assess the RBV of 25-OH-D_3_ (Zhang et al. 2018).

## Results

### Production performance

The data presented in Table 2 displays that either dietary VD_3_ or 25-OH-D_3_ supplementation increased egg production from 9 to 15 wk linearly and quadratically (*P *< 0.05). The egg production (9 to 15 wk) of breeding ducks stabilizes when the dietary VD_3_ level reaches 1,000 IU/kg or the dietary 25-OH-D_3_ level attains 250 IU/kg (*P *< 0.05). Dietary 25-OH-D_3_ supplementation of 500 IU/kg decreased the feed conversion ratio (FCR), compared with 0 IU/kg (*P *= 0.039). The egg mass in 25-OH-D_3_ group was significantly higher than that in the VD_3_ group (*P *= 0.009). However, neither dietary VD_3_ source nor levels affected the average body weight (ABW), ADFI, and egg weight of duck breeders throughout the whole trial period (*P *> 0.05).

**Table 2:** Effects of different dietary vitamin D sources and levels on body weight, feed intake, and egg production of duck breeders.

VD source	VD Level, IU/kg	ABW, kg	ADFI, g/d	FCR, g: g	Egg mass, g	Egg weight, g	Egg production, % (1 to 8 wk)	Egg production, % (9 to 15 wk)	Egg production, % (1 to 15 wk)
**VD_3_**	0	2.96	253.02	4.07	77.69	88.74	88.74	60.96^c^	76.67
	250	3.03	257.93	3.70	87.68	88.26	85.63	70.54^bc^	82.99
	500	3.15	257.46	3.53	87.31	84.90	90.21	76.72^ab^	82.80
	1000	3.07	261.04	3.51	86.32	86.35	90.23	82.38^a^	86.38
	2000	3.08	255.95	3.55	86.33	85.47	89.72	79.23^ab^	87.80
	SEM	0.341	12.482	0.683	12.787	7.366	12.560	21.250	13.087
***P*-value**	Treatment	0.563	0.388	0.091	0.504	0.371	0.365	<0.001	0.110
	Linear	0.449	0.645	0.207	0.443	0.223	0.339	<0.001	0.024
	Quadratic	0.300	0.065	0.167	0.277	0.433	0.523	0.001	0.242
**25-OH-D_3_**	0	2.96	253.02	4.07^a^	77.69	88.74	88.74	60.96^b^	76.67
	250	2.93	256.92	3.49^ab^	88.74	87.03	90.86	77.84^a^	84.90
	500	3.11	256.22	3.28^b^	88.37	85.55	88.34	77.68^a^	86.47
	1000	3.06	258.20	3.63^ab^	88.61	88.37	90.89	75.79^a^	82.01
	2000	3.21	253.70	3.39^b^	88.91	88.91	88.18	80.13^a^	85.34
	SEM	0.318	12.361	0.738	12.792	9.397	11.945	18.483	12.560
***P*-value**	Treatment	0.071	0.668	0.039	0.336	0.606	0.681	<0.001	0.148
	Linear	0.010	0.894	0.348	0.239	0.513	0.694	0.003	0.253
	Quadratic	0.819	0.159	0.757	0.211	0.648	0.426	0.027	0.333
**Comparison between VD form**									
**VD_3_**	–	3.10	91.87	3.57	86.91^b^	86.34	84.41	82.65	72.49
**25-OH-D_3_**	–	3.08	92.00	3.56	88.66^a^	87.09	87.30	80.22	75.37
** *P*-value**	–	0.744	0.927	0.881	0.009	0.476	0.206	0.425	0.374
**Comparison between supplemental VD levels**									
	250	2.96	91.35	3.60	87.46	87.61	83.43	76.42	71.96
	500	3.13	92.08	3.51	87.62	85.38	85.02	82.41	71.99
	1000	3.11	94.00	3.62	88.21	87.47	85.38	85.41	72.85
	2000	3.15	90.35	3.51	87.84	87.31	89.71	81.65	79.13
***P*-value**	–	0.056	0.367	0.608	0.859	0.317	0.222	0.199	0.334

ADFI, average daily feed intake; ABW, average body weight; VD, vitamin D; VD_3_, vitamin D_3_, 25-OH-D_3_, 25-hydroxycholecalciferol; and SEM, standard error of the mean.

a–c Means with different superscripts within the same column differ significantly (*P *< 0.05).

### Reproductive performance

The egg hatchability (7 to 10 wk) showed a significant improvement with the dietary supplementation of VD_3_ or 25-OH-D_3_ (*P *< 0.05, Table 3), and linear dose-response was observed with increasing levels of 25-OH-D_3_ (*P *= 0.011). There were no significant effects of different dietary VD sources and supplemented levels on egg fertility (1 to 6 wk and 7 to 10 wk), egg hatchability (1 to 6 wk), and duckling weight (1 to 6 wk and 7 to 10 wk) of duck breeders (*P *> 0.05).

**Table 3:** Effects of different dietary vitamin D sources and levels on reproductive performance of duck breeders.

VD source	VD Level, IU/kg	Fertility, % (1 to 6 wk)	Hatchability, % (1 to 6 wk)	Duckling weight, g (1 to 6 wk)	Fertility, % (7 to 10 wk)	Hatchability, % (7 to 10 wk)	Duckling weight, g (7 to 10 wk)
**VD_3_**	0	87.30	80.93	60.41	78.11	53.86^b^	57.62
	250	85.22	81.58	59.31	79.30	65.29^ab^	55.89
	500	90.90	87.87	59.49	87.01	64.93^ab^	56.89
	1000	85.08	82.00	59.44	82.53	75.30^a^	55.87
	2000	86.74	82.61	59.36	90.27	75.90^a^	57.58
	SEM	13.271	12.900	6.794	15.307	19.916	6.946
***P*-value**	Treatment	0.243	0.263	0.913	0.081	0.008	0.733
	Linear	0.723	0.936	0.606	0.018	0.074	0.521
	Quadratic	0.977	0.321	0.609	0.800	0.132	0.053
**25-OH-D_3_**	0	87.30	80.93	60.41	78.11	53.86^b^	57.62
	250	82.97	84.94	61.09	80.71	66.88^a^	58.69
	500	86.31	82.48	61.25	79.26	60.56^ab^	58.51
	1000	89.02	85.15	61.98	83.60	71.00^a^	57.31
	2000	83.66	83.86	58.99	84.70	68.90^a^	57.71
	SEM	14.666	12.206	8.689	18.467	18.103	5.137
***P*-value**	Treatment	0.272	0.735	0.494	0.787	0.041	0.789
	Linear	0.628	0.453	0.630	0.246	0.011	0.622
	Quadratic	0.275	0.393	0.112	0.782	0.380	0.977
**Comparison between VD form**							
**VD_3_**	–	87.00	83.58	59.32	84.78	70.50	56.15^b^
**25-OH-D_3_**	–	85.53	84.00	60.32	82.01	66.84	58.05^a^
** *P*-value**	–	0.339	0.829	0.106	0.248	0.565	0.005
**Comparison between supplemental VD levels**							
	250	84.08	82.95	60.21	80.00	67.06	57.31
	500	88.66	85.55	60.93	83.13	62.81	56.80
	1000	87.07	83.52	59.15	83.05	73.22	56.59
	2000	85.23	83.18	59.17	87.57	75.90	57.64
***P*-value**	–	0.177	0.802	0.130	0.163	0.053	0.7073

VD, vitamin D; VD_3_, vitamin D_3_; 25-OH-D_3_, 25-hydroxycholecalciferol; and SEM, standard error of the mean.

a–b Means with different superscripts within the same column differ significantly (*P *< 0.05).

### Egg quality

Egg Haugh unit was increased linearly as the dietary level of VD_3_ increased (*P *= 0.021, Table 4) whereas increased quadratically as the dietary level of 25-OH-D_3_ increased (*P *= 0.042). The yolk color in ducks fed diets containing 1,000 IU/kg of VD was observed to be lower, irrespective of the source of VD (*P *= 0.005). Dietary VD_3_ or 25-OH-D_3_ supplemented levels did not affect egg albumen height, eggshell breaking strength, and thickness (*P *> 0.05).

**Table 4:** Effects of different dietary vitamin D sources and levels on egg quality of duck breeders.

VD source	VD level, IU/kg	Eggshell breaking strength, kgf	Eggshell thickness, mm	Yolk color	Albumen height, mm	Haugh unit
**VD_3_**	0	3.26	0.332	4.44	5.17	56.08^c^
	250	3.18	0.354	4.52	6.33	73.30^ab^
	500	3.19	0.349	4.32	5.59	64.52^bc^
	1000	3.61	0.347	4.11	5.89	68.75^ab^
	2000	3.18	0.335	4.33	6.90	78.22^a^
	SEM	1.129	0.0517	0.844	2.138	16.783
***P*-value**	Treatment	0.413	0.660	0.417	0.112	0.007
	Linear	0.912	0.724	0.521	0.019	0.021
	Quadratic	0.373	0.384	0.327	0.959	0.547
**25-OH-D_3_**	0	3.26	0.332	4.44	5.17	56.08^b^
	250	3.53	0.353	4.66	6.27	73.57^a^
	500	3.39	0.364	4.37	5.81	72.14^a^
	1000	3.01	0.353	4.13	5.88	68.82^a^
	2000	3.56	0.348	4.29	6.12	66.17^a^
	SEM	1.101	0.0474	0.935	1.983	14.327
***P*-value**	Treatment	0.227	0.550	0.247	0.598	0.019
	Linear	0.693	0.744	0.278	0.238	0.554
	Quadratic	0.347	0.202	0.437	0.454	0.042
**Comparison between VD form**						
**VD_3_**	–	3.30	0.346	4.31	6.17	69.24
**25-OH-D_3_**	–	3.39	0.355	4.32	5.97	68.18
***P*-value**	–	0.453	0.193	0.942	0.387	0.636
**Comparison between supplemental VD levels**						
	250	3.36	0.354	4.59^a^	6.30	72.22
	500	3.30	0.358	4.36^a^	5.75	66.97
	1000	3.33	0.350	4.01^b^	5.86	66.00
	2000	3.40	0.342	4.31^ab^	6.49	70.18
***P*-value**	–	0.969	0.402	0.005	0.074	0.141

VD, vitamin D; VD_3_, vitamin D_3_; 25-OH-D_3_, 25-hydroxycholecalciferol; SEM, standard error of the mean.

a–c Means with different superscripts within the same column differ significantly (*P *< 0.05).

### Ovarian follicle development

Table 5 shows the impact of dietary VD_3_ or 25-OH-D_3_ levels on ovarian follicle development of duck breeders. Both dietary VD_3_ and 25-OH-D_3_ significantly increased the number and weight of dominant follicle, ovary weight, and ovary index of duck breeders (linear and quadratic, *P *< 0.05) compared to the control group. The weight and number of large yellow follicles increased quadratically with rising dietary VD_3_ levels (*P *< 0.05), while they exhibited both linear and quadratic increases with higher dietary 25-OH-D_3_ supplementation (*P *< 0.05). Duck breeders consuming diets supplemented with 25-OH-D_3_ displayed greater ovary weight and an increased number and weight of dominant follicles compared to those supplemented with VD_3_ (*P *< 0.05).

**Table 5:** Effects of different dietary vitamin D sources and levels on ovarian and follicle development of duck breeders.

VD source	VD level, IU/kg	Ovarian weight, g	Ovarian index, %	Dominant follicle weight, g	Dominant follicle number	Large yellow follicle weight, g	Large yellow follicle number
**VD_3_**	0	21.40^b^	0.69^b^	12.50^b^	1.87^b^	0.72^b^	2.67^b^
	250	59.10^a^	1.94^a^	48.00^a^	3.92^a^	2.13^a^	10.00^a^
	500	58.67^a^	1.87^a^	45.95^a^	3.92^a^	1.83^ab^	9.33^a^
	1000	68.19^a^	2.24^a^	55.56^a^	4.93^a^	2.39^a^	11.33^a^
	2000	61.95^a^	2.02^a^	52.21^a^	4.75^a^	1.77^ab^	7.41^a^
	SEM	28.254	0.916	27.123	2.264	1.382	5.689
***P*-value**	Treatment	<0.001	<0.001	<0.001	0.004	0.020	0.001
	Linear	0.008	0.007	0.006	0.007	0.226	0.278
	Quadratic	0.001	<0.001	0.004	0.020	0.010	<0.001
**25-OH-D_3_**	0	21.40^b^	0.69^b^	12.51^b^	1.87^b^	0.72^b^	2.67^b^
	250	71.82^a^	2.39^a^	57.55^a^	5.17^a^	1.62^ab^	7.25^ab^
	500	71.51^a^	2.17^a^	60.21^a^	5.25^a^	2.26^a^	8.00^ab^
	1000	72.31^a^	2.39^a^	58.10^a^	5.29^a^	2.98^a^	12.87^a^
	2000	80.00^a^	2.53^a^	70.83^a^	5.33^a^	2.69^a^	9.50^a^
	SEM	25.054	0.819	22.956	1.752	1.704	7.189
***P*-value**	Treatment	<0.001	<0.001	<0.001	<0.001	0.005	0.007
	Linear	<0.001	<0.001	0.006	0.001	0.006	0.023
	Quadratic	<0.001	<0.001	0.004	<0.001	0.013	0.006
**Comparison between VD form**							
**VD_3_**	–	62.34^b^	2.03	50.73^b^	4.41^b^	2.05	9.63
**25-OH-D_3_**	–	73.81^a^	2.37	62.14^a^	5.20^a^	2.42	9.61
***P*-value**	–	0.036	0.060	0.033	0.038	0.273	0.934
**Comparison between supplemental VD levels**							
	250	65.56	2.16	54.22	4.54	1.87	8.63
	500	65.09	2.02	53.08	4.58	2.04	8.67
	1000	70.25	2.32	56.83	5.00	2.69	12.10
	2000	70.97	2.27	61.52	5.04	2.11	8.46
***P*-value**	–	0.808	0.645	0.698	0.682	0.260	0.158

VD, vitamin D; VD_3_, vitamin D_3_; 25-OH-D_3_, 25-hydroxycholecalciferol; and SEM, standard error of the mean.

a–b Means with different superscripts within the same column differ significantly (*P *< 0.05).

### Plasma biochemical indicators

In comparison to the control group, dietary 25-OH-D_3_ supplementation increased plasma P (*P *= 0.002, Table 6) and Ca contents (linear, *P *= 0.001). A linear increase in plasma 25-OH-D_3_ concentration was observed with elevated dietary levels of VD_3_ or 25-OH-D_3_ (*P *< 0.05). Additionally, ducks fed with 25-OH-D_3_ had higher plasma 25-OH-D_3_ contents than those fed with VD_3_ (*P *< 0.001), with the highest levels recorded in the group receiving 2000 IU/kg (*P *< 0.001). However, there were no effects of different dietary VD sources and supplemented levels on the plasma Ca and P contents of ducks (*P *> 0.05).

**Table 6:** Effects of different dietary vitamin D sources and levels on plasma biochemical indices of duck breeders.

VD source	VD level, IU/kg	Ca, mmol/L	P, mmol/L	25-OH-D_3_, ng/mL
**VD_3_**	0	3.97	2.04	113.96^b^
	250	6.12	3.30	145.54^b^
	500	6.14	2.98	167.51^b^
	1000	6.75	3.28	229.44^a^
	2000	5.90	3.30	272.43^a^
	SEM	2.457	1.043	36.802
***P*-value**	Treatment	0.054	0.111	<0.001
	Linear	0.193	0.011	<0.001
	Quadratic	0.031	0.247	0.120
**25-OH-D_3_**	0	3.97^b^	2.04^b^	113.96^d^
	250	6.72^a^	3.51^a^	169.31^cd^
	500	6.96^a^	3.34^a^	254.10^bc^
	1000	6.29^a^	2.95^a^	295.91^b^
	2000	7.54^a^	3.64^a^	428.40^a^
	SEM	1.949	1.345	70.671
***P*-value**	Treatment	<0.001	0.002	<0.001
	Linear	0.001	0.084	<0.001
	Quadratic	0.072	0.125	0.243
**Comparison between VD form**				
**VD_3_**	–	6.21	3.22	205.24^b^
**25-OH-D_3_**	–	6.87	3.35	280.78^a^
** *P*-value**	–	0.122	0.563	<0.001
**Comparison between supplemental VD levels**				
	250	6.63	3.42	159.80^c^
	500	6.36	3.17	219.47^b^
	1000	6.51	3.11	265.70^b^
	2000	6.72	3.47	359.08^a^
***P*-value**	–	0.917	0.631	<0.001

VD, vitamin D; VD_3_, vitamin D_3_; 25-OH-D_3_, 25-hydroxycholecalciferol; and SEM, standard error of the mean.

a–d Means with different superscripts within the same column differ significantly (*P *< 0.05).

### Estimation of the requirement of VD_3_ and 25-OH-D_3_ in diet

The estimated requirements for VD_3_ and 25-OH-D_3_ in aged breeding ducks, derived from quadratic regression analyses of reproductive performance characteristics, are summarized in Table 7. For duck breeders (64 to 79 wk of age), the VD_3_ requirements were estimated to be 906, 359, and 730 IU/kg for egg production, ovarian weight, and dominant follicle number, respectively, whereas the 25-OH-D_3_ requirements were 260, 324, and 308 IU/kg, respectively.

**Table 7:** Estimation of the dietary vitamin D requirements for duck breeders fed diet based on quadratic regression analysis.

Variables	VD source	Regression equation	Requirement, IU/kg	*R* ^2^	*P*-value
**Egg production (9 to 15 wk)**	VD_3_	y = 80.79-0.00002(906.2-x)^2^	906	0.983	0.017
	25-OH-D_3_	y = 77.87-0.00025(260.3-x)^2^	260	0.960	0.040
**Ovarian weight**	VD_3_	y = 62.94-0.00032(359.2-x)^2^	359	0.966	0.034
	25-OH-D_3_	y = 74.61-0.00051(324.2-x)^2^	324	0.981	0.019
**Dominant follicle number**	VD_3_	y = 4.79-0.00002(836.5-x)^2^	730	0.943	<0.001
	25-OH-D_3_	y = 5.29-0.00004(307.6-x)^2^	308	0.999	<0.001

VD: vitamin D; VD_3_: vitamin D_3_; 25-OH-D_3_: 25-hydroxycholecalciferol.

1 Y is the dependent variable and X is the dietary VD level (IU/kg).

2 Dietary VD requirement = X giving 95% of the maximal response (IU/kg).

### Relative bioavailability estimates of 25-OH-D_3_

As shown in Table 8, based on the daily dietary VD_3_ and 25-OH-D_3_ intake added during the experiment, the regressions within the linear range were calculated by broken-line analysis. Relationships characterized by multiple linear regression were observed among ovarian weight, the number of dominant follicles, and plasma levels of 25-OH-D_3_ (*P *< 0.05). Thus, these parameters of daily dietary VD_3_ and 25-OH-D_3_ intake were used to estimate the RBV (Table 9). VD_3_ and 25-OH-D_3_ slopes varied greatly regarding ovarian weight, number of dominant follicles, and plasma 25-OH-D_3_ concentration (*P *< 0.05). With VD_3_ response at 100%, the RBV of 25-OH-D_3_ was estimated to be 147%, 191%, and 211% using the mentioned indices above, respectively.

**Table 8:** Multiple linear regressions of egg production, ovarian weight, dominant follicle number and plasma 25-OH-D_3_ on daily dietary vitamin D_3_ intake.^1^

Response criterion	Multiple linear regression equation^2^	*R* ^2^	*P-*value
**Ovarian weight**	*Y *= 32.127 + 0.368 *X_1_* +0.2499 *X_2_*	0.2129	<0.001
**Dominant follicle number**	*Y *= 2.5026 + 0.0253 *X_1_* +0.0132 *X_2_*	0.1986	0.001
**Plasma 25-OH-D_3_**	*Y *= 139.1 + 0.5893 *X_1_* +0.2791 *X_2_*	0.7018	<0.001

25-OH-D_3_: 25-hydroxycholecalciferol.

1 Daily dietary vitamin D_3_ intake = ADFI times supplemental dietary vitamin D_3_ levels for each respective vitamin D_3_ source.

2 *X_1_* is the daily added vitamin D_3_ intake (IU) from 25-OH-D_3_, and *X_2_* is the daily added vitamin D_3_ intake (IU) from vitamin D_3_.

**Table 9:** RBV of 25-OH-D_3_ based on slope ratios from multiple linear regressions of ovarian weight, dominant follicle number, and plasma 25-OH-D_3_ on daily dietary vitamin D intake.^1^

Response criterion	VD source	Regression coefficients		RBV		*P-*value^2^
		Slope	SE	%	SE	
**Ovarian weight**	VD_3_	0.2499	0.0933	100	0.48	<0.001
	25-OH-D_3_	0.3680	0.0928	147		
**Dominant follicle number**	VD_3_	0.0132	0.0066	100	0.81	0.016
	25-OH-D_3_	0.0253	0.0066	191		
**Plasma 25-OH-D_3_**	VD_3_	0.2791	0.0633	100	0.43	<0.001
	25-OH-D_3_	0.5893	0.0593	211		

VD, vitamin D; VD_3_, vitamin D_3_; 25-OH-D_3_, 25-hydroxycholecalciferol; and RBV, relative bioavailability values; SE, standard error.

1 Daily dietary vitamin D_3_ intake = ADFI times supplemental dietary vitamin D_3_ levels for each respective vitamin D_3_ source.

2 *P-*value for the difference in slopes among vitamin D sources.

## Discussion

VD_3_ contributes to the growth and development of both livestock and poultry (Adhikari et al. 2020; Zhuang et al. 2025). Under the intensive farming mode, duck exposure to direct sunlight ios limited and less VD_3_ is synthesized from dehydrocholesterol in the skin, thus, it is necessary to supplement VD_3_ in diet (Farquharson and Jefferies 2000). Geng et al. (2018) found that dietary VD_3_ supplementation increased egg production of laying hens. Jin et al. (2024) demonstrated that dietary supplementation with 25-OH-D_3_ not only increased egg production but also reduced the FCR in laying ducks. Furthermore, after adding 25-OH-D_3_ to the diet for 12 wk, the egg-laying rate of laying hens reached the maximum (Gao et al. 2024). In alignment with earlier research, in our study, with the increase of dietary VD_3_ or 25-OH-D_3_ levels, egg production (9 to 15 wk) of ducks was increased. Furthermore, the inclusion of 25-OH-D_3_ notably improved the FCR. Thus, VD_3_ and 25-OH-D_3_ can actively improve egg production of poultry, which may be related to their ability to promote follicle development (Hrabia et al. 2023), and the similar findings were also observed in this study.

It has been shown that VD_3_ deficiency impaired ovarian function and decreased reproductive performance (Abuzeid 2020), whereas dietary VD_3_ supplementation promoted follicle development, especially dominant follicle (Kim et al. 2014). In alignment with earlier research, in our study, supplementation of dietary VD_3_ or 25-OH-D_3_ increased the ovaries, ovarian index, dominant follicle weight, and dominant follicle number. VD_3_ and its metabolites (25-OH-D_3_, 1α,25-Dihydroxyvitamin D_3_) promote cell cycle progression from the G1 phase to the S phase and subsequently to the G2/M phase by downregulating the cell cycle inhibitor *cyclin-dependent kinase inhibitor 2D* and upregulating genes, such as *thrombomodulin*, thereby effectively stimulating granulosa cell proliferation (Chen et al. 2021; Hu et al. 2023; Cheng et al. 2023). Granulosa cells, which are the primary cells surrounding the oocyte within the follicle, play a crucial role in follicular development (Zhang et al. 2024). The rapid proliferation of granulosa cells facilitates the transition of follicles into antral follicles, leading to their maturation (Zhu et al. 2019). Furthermore, 25-OH-D_3_ contributes to a healthier microenvironment for follicular development by mitigating ovarian oxidative stress and inflammation (Berry et al. 2022). The quality of follicular development determines the reproductive performance of poultry. Our results indicated that the administration of dietary VD_3_ or 25-OH-D_3_ in laying duck breeders improved the egg-laying rate by promoting ovarian function. Nonetheless, dietary supplementation with either VD_3_ or 25-OH-D_3_ did not show a significant effect on ABW, ADFI, and egg weight of the duck breeders (64 to 79 wk of age), which is consistent with previous studies (Adhikari et al. 2020). The addition of 25-OH-D_3_ did not impact the body weight of laying ducks and laying hens (Silva 2017; Li et al. 2023; Jin et al. 2024). Hence, neither VD_3_ nor 25-OH-D_3_ affects body weight and ADFI of aged poultry.

Sufficient maternal VD is crucial for embryonic development. The duck breeders fed the basal diet without VD supplementation showed the lowest hatchability among all groups in our study. This aligns with earlier research indicating that a lack of VD in hatching eggs results in increased mortality rates among embryos (Sunde et al. 1978; Stevens et al. 1984; Elaroussi et al. 1993). Similarly, providing water with 34.5 μg of 25-OH-D_3_ per liter for broiler breeders lowered the rate of early embryonic mortality from 6.22% to 4.37% when contrasted with the control group (Saunders-Blades and Korver 2015). The development of a duck embryo depends on nutrients deposited in the eggs. The lack of VD_3_ in the maternal ducks would reduce the VD_3_ content in embryonic eggs, thereby depressing embryonic development (Waters et al. 2001). Dietary VD_3_ supplementation would increase the VD_3_ content in egg yolk (Yao et al. 2013; Wen et al. 2019) and 25-OH-D_3_ or 1,25-Dihydroxyvitamin D_3_ content in the embryo, and therefore promote embryonic development and hatchability (Chen et al. 2021).

In this study, the administration of VD_3_ or 25-OH-D_3_ in the duck breeders increased the egg Haugh unit. Laying hens fed a diet of 25-OH-D_3_ supplementation saw an increase in egg Haugh unit (Chen et al. 2020). The Haugh unit represents egg freshness (Williams 1992), and the decrease in protein quality in the egg white leads to a reduction in the Haugh unit (Zhou et al. 2021). In studies on pregnant sows (Li et al. 2023), 25-OH-D_3_ promotes the amino acid transport between the mother and the fetus by upregulating genes, such as sodium-dependent neutral amino acid transporter 2 and L-type amino acid transporter 1. This mechanism may also occur during the follicular development stage of poultry, facilitating the synthesis and transport of protein precursors, and thus increasing the Haugh unit of poultry eggs. Furthermore, 25-OH-D_3_ could directly bind to the VD receptor and regulate the expression of ovalbumin genes, thereby improving the protein quality in egg white (Li et al. 2023). These results indicated that dietary VD_3_ or 25-OH-D_3_ supplementation contributed positively to the egg white quality. In this study, dietary supplementation with 1,000 IU/kg of VD significantly reduced yolk color. The color of egg yolk is primarily determined by fat-soluble pigments that poultry ingest from their feed, the most important of which are carotenoids. After absorption in the intestine, these pigments are transported to the liver via the bloodstream, incorporated into very low-density lipoproteins, and ultimately delivered to developing follicles, where they are deposited in the yolk (Chang Y et al. 2024). Previous studies have not observed significant changes in yolk color due to varying levels of VD_3_ (Persia et al. 2013; Ren et al. 2016; Likittrakulwong et al. 2021; Romero et al. 2024), which is inconsistent with the results of the current experiment. In addition, in our study, the breaking strength and thickness of eggshell didn’t change by dietary VD_3_ or 25-OH-D_3_ adding. Research indicated that the influence of VD_3_ on eggshell quality is associated with the levels of dietary Ca (Attia et al. 2020). The addition of diets containing 3.5% Ca along with 1,000 IU of VD_3_ or a total of 4,000 IU/kg VD_3_ enhanced the performance of hens that were fed a diet with 3.5% Ca during the later stages of production (60 to 72 wk of age), demonstrating that the effects of VD_3_ are contingent on dietary Ca concentrations (Attia et al. 2020). Interestingly, whether VD administration can improve eggshell quality remains unclear, as previous studies have shown mixed results. Silva (2017) found that 25-OH-D_3_ (2760 IU/kg) supplementation throughout the laying cycle in hens improved the eggshell thickness. Compared with commercial supplementary doses of VD_3_ (2500 IU/kg), the high supplementary doses of VD_3_ or 25-OH-D_3_ (5000 IU/kg) improved strength and thickness of eggshell in aged laying hens (Jing et al. 2022). Conversely, at 70 wk of age, 25-OH-D_3_ administration (69 μg/kg or 125 μg/kg) did not affect eggshell quality (Li et al. 2023). This may be due to different bird strains and trial periods.

The plasma levels of 25-OH-D_3_ are often a gauge of VD status of animals (Backus and Foster 2021, McKenna and Kilbane 2022). In this study, the content of 25-OH-D_3_ in plasma was increased linearly with the increasing dietary level of VD_3_ or 25-OH-D_3_. Similar phenomena had been reported for pigs, yellow-feathered broilers, and starter Pekin ducks (Jiang et al. 2015; Burild et al. 2016; Zhang et al. 2021; Tang et al. 2025). In addition, the plasma levels of 25-OH-D_3_ in the group receiving 25-OH-D_3_ supplementation exceeded those in the VD_3_ groups in this study. This observation aligns with earlier research indicating that incorporating 25-OH-D_3_ into the diet results in elevated plasma 25-OH-D_3_ concentrations compared to VD_3_ (Soares et al. 1995; Han et al. 2016; Zhang et al. 2021). The incorporation of 25-OH-D_3_ into the diet not only bypassed the metabolic pathway of the liver, but also had better fat solublity (Jones 2008). Therefore, compared with VD_3_, incorporating 25-OH-D_3_ into the diet reduces conversion loss and is easier utilized. In our experiments, the plasma Ca and P levels in ducks were significantly elevated in the 25-OH-D_3_ treatment group, whereas no such increase was observed in the VD_3_ treatment group. This phenomenon may be attributed to the enhanced bioavailability of 25-OH-D_3_. Previous studies have demonstrated that dietary supplementation with 25-OH-D_3_ significantly raises plasma Ca and P concentrations in layer ducks during the late laying period, which is accompanied by an upregulation of intestinal Ca and P transport-related genes (Zhang et al. 2025). The observed increase in plasma Ca and P levels is advantageous for sustaining optimal laying performance, eggshell quality, and skeletal health in breeding ducks during the late laying period (Liu et al. 2023; Hu et al. 2025; Uyanga et al. 2025; Zhang et al. 2025).

The suggested amount of VD_3_ for the diets of ducklings is between 200 IU/kg and 400 IU/kg (Fritz et al. 1941; Tang et al. 2025). However, relevant data regarding the VD_3_ requirements in duck breeders remain insufficient. The NRC (1994) recommends VD_3_ intake of 900 IU/kg for duck breeders. And our study indicated that the VD_3_ requirement for duck breeders during the late egg-laying period were 359 to 906 IU/kg, which is close to the NRC (1994) recommendation. Furthermore, during the laying peak period, the hens’ demand for VD_3_ and 25-OH-D_3_ distance was 254.1 IU/kg or 146.7 IU/kg (Li et al. 2023). The research suggests that the ideal dietary need for 25-OH-D_3_ is less than that of VD_3_, a finding that was similarly observed in young White Pekin ducks (Tang et al. 2025). In this study, dietary VD_3_ requirements of ducks (64 to 70 wk of age) were 359 ∼ 906 IU/kg, whereas the 25-OH-D_3_ requirements were 260 ∼ 324 IU/kg. Egg production is recommended as the evaluation criterion. Therefore, supplementing the basal diet with 906 IU/kg VD_3_ or 324 IU/kg 25-OH-D_3_ is recommended for duck breeders (64 to 70 wk of age).

As a derivative of VD_3_ in the liver, Zhou et al. (2022) found that 25-OH-D_3_ had higher bioavailability than VD_3_. The RBV of 25-OH-D_3_ to VD_3_ associated with improved growth performance and bone mineralization of broiler chicken from 1 to 21 days was 2.03 (Han et al. 2016). Furthermore, the RBV of 25-OH-D_3_ to VD_3_ was 1.38 and 1.33 in broiler breeders based on daily egg production and hatchability, respectively (Atencio et al. 2005). However, there is still a lack of relevant reports on duck breeders. In our study, based on egg production, ovarian weight, dominant follicle number, and the 25-OH-D_3_ level in plasma, the RBV of 25-OH-D_3_ in comparison to VD_3_ ranged from 147% to 211%. Earlier findings in broilers suggest these results are similar (Atencio et al. 2005; Han et al. 2016). This phenomenon may be due to the higher absorption, transport, and metabolic efficiency of 25-OH-D_3_. It exhibits a greater affinity for the VD-binding protein in intestinal cells and can more effectively penetrate the bloodstream via intestinal mucosal cells (McDowell 2008). Additionally, 25-OH-D_3_ can bypasses the 25-hydroxylation reaction in the liver, which makes its utilization in the body more efficient than VD_3_ (Lütke‐Dörhoff et al. 2022).

## Conclusion

Supplemental VD_3_ or 25-OH-D_3_ provided in the diets of duck breeders could increase the egg production, hatchability, Haugh unit, and 25-OH-D_3_ level in plasma. Additionally, the efficacy of 25-OH-D_3_ in enhancing ovarian weight, ovarian index, both the number and weight of dominant follicle, and 25-OH-D_3_ plasma content surpassed that of VD_3_. Supplementing the basal diet with 906 IU/kg VD_3_ or 324 IU/kg 25-OH-D_3_ is recommended for duck breeders (64 to 70 wk of age), and the bioavailability of 25-OH-D_3_ relative to VD_3_ is 211%.

## Abbreviations

ADFI: average daily feed intake
ABW: average body weight
AME: average metabolizable energy
Ca: calcium
FCR: feed conversion ratio
GLM: generalized linear models
P: phosphorus
RBV: relative bioavailability
SEM: standard error of the mean
NRC: national research council
VD_3_: vitamin D_3_
VD: vitamin D
25-OH-D_3_: 25-hydroxycholecalciferol
